# Electron beam irradiation induced crystallization behavior of amorphous Ge_2_Sb_2_Te_5_ chalcogenide material

**DOI:** 10.1186/s42649-019-0021-5

**Published:** 2019-12-17

**Authors:** Byeong-Seon An

**Affiliations:** School of Advanced Materials Science & Engineering, Sungkyunkwan University, Suwon, 16419 South Korea

**Keywords:** Ge-Sb-Te based chalcogenide, Transmission electron microscopy, Electron beam irradiation, Crystallization

## Abstract

The crystallization of amorphous Ge_2_Sb_2_Te_5_ phase change material induced by electron beam irradiation was investigated by in-situ transmission electron microscopy (TEM). Amorphous matrix transformed into a partially crystalline state after being irradiated with a 200-keV electron beam for a long time. Real-time observation revealed that the crystallization of amorphous Ge_2_Sb_2_Te_5_ film occurs through a nucleation and growth mechanism under electron beam irradiation in TEM. While uncertainty from the 2D projection remains, the nuclei have been observed to grow preferentially along the < 100> direction.

Transmission electron microscopy (TEM) is a powerful tool for analyzing phase change materials and provides localized information about the microstructures. However, high energy electron beam irradiation may affect the kinetics of phase transformation in the chalcogenide materials (Nagase et al., 2004; Kooi et al., 2004; Zhou et al., 2018). (Zhou et al., 2018) investigated the phase change mechanism for chalcogenide materials using in-situ heating TEM and selected area electron diffraction, and reported that Ge_2_Sb_2_Te_5_ is a nucleation-dominated material, while Si_2_Sb_2_Te_3_ and Ti_0.5_Sb_2_Te_3_ are growth-dominated materials. However, the electron beam irradiation induced crystal growth behavior such as a preferred growth direction of crystalline Ge_2_Sb_2_Te_5_ has not been reported.

In this work, the crystal growth behavior of amorphous Ge_2_Sb_2_Te_5_ material induced by electron beam irradiation is demonstrated using in-situ TEM (JEOL JEM-ARM200F) operated at 200 kV. The figure shows high-resolution TEM (HRTEM) images and Fast Fourier Transformed (FFT) diffractograms in the inset for the amorphous Ge_2_Sb_2_Te_5_ film subjected to 200 keV electron beam irradiation. The HRTEM images show the microstructural evolution of amorphous Ge_2_Sb_2_Te_5_ film after electron beam irradiation for t = 0, 10, 24, 40, 41, 45, and 48 min, where t refers to the time elapsed after the start of electron beam irradiation. Firstly, initial Ge_2_Sb_2_Te_5_ film was amorphous phase, which was confirmed by a halo-ring pattern in FFTs as shown in Fig. 1a. After the electron beam irradiation for 10 min, small nuclei with a diameter of 6.0 nm was observed in the Ge_2_Sb_2_Te_5_ film and few diffracted spots were simultaneously found in the FFTs (Fig. 1b). The nucleus indicated by the red arrow was observed to grow in a regular atomic arrangement by electron beam irradiation for a period of 24 to 48 min (Fig. 1c-g). From direct observation using in-situ TEM, it was confirmed that the crystallization of the Ge_2_Sb_2_Te_5_ film induced by electron beam irradiation was a nucleation-dominated process, consistent with the previous work (Zhou et al., 2018). Further HRTEM analysis revealed the lattice structure and the preferred growth direction of the crystallized grains. As shown in figure (Fig. 1h), facets were developed in front of the growing fcc crystal grain and were parallel to {110} planes according to the result of phase analysis using HRTEM image and FFT in the inset. However, 2D projection of a cube in the < 111> direction can create 6 cube edges which are parallel to the {110} planes as shown in figure (Fig. 1i). The facets can be either {100} or {110} because of the projection artifacts. In other words, the determination of the growth direction based on a single electron diffraction pattern or HRTEM image may leave some degree of uncertainty. Nevertheless, the facet is most likely to be {100} rather than {110} due to the lower surface energy of the {100} plane.

**Fig. 1:**
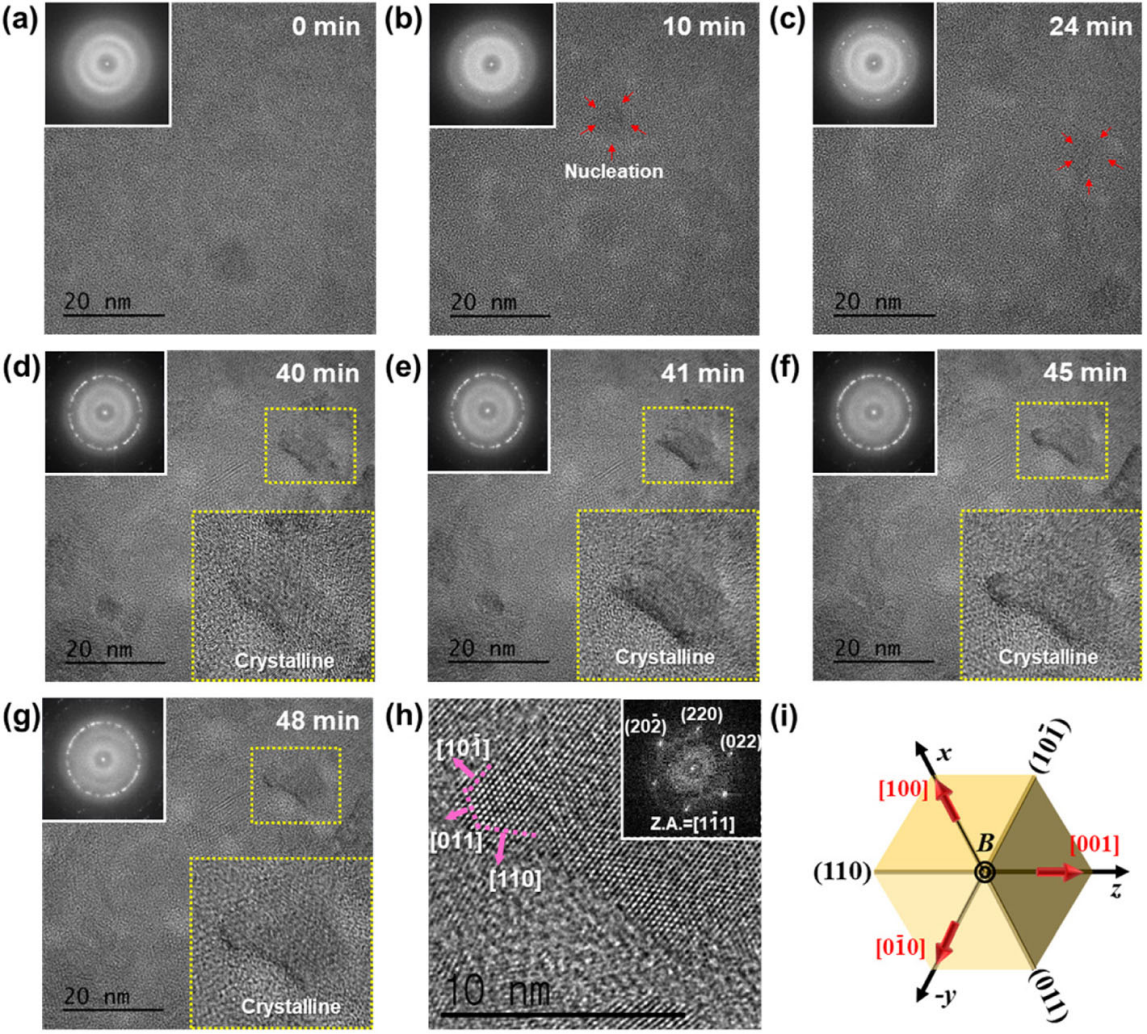
Real-time TEM images and FFTs diffractograms in the inset for the amorphous Ge_2_Sb_2_Te_5_ film during 200 keV electron beam irradiation

Consequently, crystallization of amorphous Ge_2_Sb_2_Te_5_ film occurs through a nucleation dominated mechanism under electron beam irradiation in TEM, and the nuclei preferentially grow along the < 100> direction, developing {100} facets.

## Data Availability

Not applicable.
